# STRATEGIES FOR INVOLVING OLDER ADULTS AND INFORMAL CAREGIVERS IN HEALTH POLICY DEVELOPMENT: A SCOPING REVIEW

**DOI:** 10.1093/geroni/igad104.0175

**Published:** 2023-12-21

**Authors:** Opeyemi Kolade, Porat-Dahlerbruch Joshua, Rustem Makhmutov, Theo van Achterberg, Moriah Ellen

**Affiliations:** Ben-Gurion University of the Negev, Midreshet, HaDarom, Israel; University of Pittsburgh, Pittsburgh, Pennsylvania, United States; Martin Luther Universität Halle-Wittenberg, Halle, Sachsen-Anhalt, Germany; KU Leuven, Leuven, Vlaams-Brabant, Belgium; Ben Gurion University of the Negev, Beer Sheva, HaDarom, Israel

## Abstract

Encouraging the active involvement of older adults and informal caregivers in policymaking can lead to cost-effective health and long-term care interventions and enhance the creation of responsive policies. As part of the European Union TRANS-SENIOR program, a scoping review was conducted using the Joanna Briggs Institute’s methodology. Published and grey literature was searched. Studies were included if they described the use/evaluation of methods for engaging older adults (65 years and above) and informal caregivers in local, national, or regional health policy development. Analysis was guided by the Multidimensional Framework For Patient And Family Engagement In Health And Health Care. Thirteen engagement strategies were identified from 11 publications meeting the inclusion criteria. Adopting the World Bank’s categorization of citizen engagement methods, strategies were categorized as traditional, deliberative, and others. Older adults and informal caregivers are often consulted to elicit opinions for agenda/priority setting. However, their involvement in other stages of the policy cycle: health policy formulation, implementation, and evaluation, was unclear from the literature. The described engagement methods suggested that older adults and their informal caregivers do not often have shared equal power and shared responsibility in policymaking. Findings will guide policymakers in identifying and incorporating engagement strategies to support evidence-informed policymaking processes that can improve health outcomes for older adults/informal caregivers. Future research is needed on the evaluation of involvement methods.

